# Notes and News

**Published:** 1901-03-16

**Authors:** 


### NOTES AND NEWS.

The Assistance Publique of Paris are considering the adoption of motor cars for ambulance purposes.

Sir Douglas Powell and Sir Thomas Barlow have been created Knight Commanders of the Royal Victorian Order.

Dr. Daniel C. Gilman, who has been with the Johns Hopkins University since its foundation, has resigned his post of President of the University.

Mr. Brodrick, in dealing with Army Reform in the House of Commons, has made a declaration that he includes the Army Medical Service. It is to be hoped that in any reforms he may introduce he will recognise that even in time of peace the service is under-manned.

Changes in the pattern of hospital tents are under consideration by the military authorities, and attention is being drawn to the methods of conveying water for the troops. Lord Dundonald has introduced a water cart which is designed to mechanically prevent the collection of sediment.

Another medical pilgrimage to the watering-places of France is being arranged for September. The travellers will proceed under the direction of Professor Landouzy. The places to be visited are situated in the South-east of France, and include Eocari, Devonne, St. Gervais, Sanatorium d'Hautville Aix, Marlioz, Challes, Salin-Moutiers Brides, Allevard, Uriage, La Mette Vals and Lamalou. All particulars are to be had from M. le Docteur, Carron dc la Carrierè, 2 Rue Lincoln, Paris.

The plan introduced by Surgeon-General Stephenson for distributing the supply of milk obtainable at Pretoria amongst the military hospitals appears to be an excellent one. The supply is not equal to the demand, and it is therefore especially necessary that there should be a fair distribution and that the special wants of enteric and fever cases should be supplied first. For this purpose all the milk goes to a central depôt, it is there sterilised and distributed to the hospitals in amounts varying according to the daily needs which are reported by each hospital to the depôt.

The rate of infant mortality is exceedingly high in France, amounting of late years to 317 per thousand births.

The St. Patrick's festival and dinner of the Irish Medical Schools, will take place on Saturday, 16th inst., at the Hotel Cecil.

A physician of Brooklyn, in the United States, has come off very lightly with a fine of £20 for falsifying the death certificate of a man who died of small-pox.

The Annual Meeting of the Royal National Hospital for Consumption at Ventnor will be held at the office, 34 Craven Street, Charing Cross, on March 28th at 4.30 p.m.

The Professorship of Surgery in the Royal College of Surgeons of Ireland will be filled on April 18th. The post is terminable yearly, but the holders are open to re-election.

The Annual Meeting of the Dental Hospital of London will be held at the new hospital in Leicester Square on Thursday, March 28th, at 5.30 p.m., Stuart Samuel, Esq., M.P., presiding.

The shilling subscription list which has been opened by the *Cardiff Western Mail* has resulted in the large sum of £5,375, for the benefit of the Cardiff Infirmary. Such a result does great credit to the energies of the local press.

A terrible fatality is reported from Tokio, where the hospital of the Medical Department of the University—a wooden structure—was destroyed by fire. No less than twenty-one patients lost their lives, and ten nurses and patients were seriously injured.

The most thorough investigation is being made of the plague-infected districts at the Cape. The Kaffir population had succeeded in secreting a certain number of cases, but the tainted houses were surrounded by the police, and the infected persons and contacts removed. All the public-houses have been closed in the Kaffir quarter, and the Kaffirs are shortly to be removed to another locality.

The legal contention in American courts that Christian Scientists are not of sound mind will not be very pleasant hearing for the fashionable people who have taken up this latest novelty in religions. In America the question has arisen over the validity of the will of a Christian Scientist. The weight of medical evidence is strongly against the Scientists, who are considered by the profession to be suffering from delusions.

The beautiful new Home and Hospital for Jewish Incurables is now nearly completed at South Tottenham. The style of architecture is Elizabethan; red brick, terra cotta and stone are employed in the structure. On the ground floor are four dormitories for six or more beds, two single wards, large entertainment hall, two sitting-rooms, and secretary's and matron's rooms. The second floor is arranged much in the same way, whilst the third floor is devoted to the housing of the nurses and domestic staff, and contains the kitchen and domestic offices. Lifts connect the kitchen with the service room. The lavatories and bath-rooms are in an annexe, the baths being sunk in the floor to be more readily utilised for helpless patients. The Institution has been built and furnished for £27,000.

